# The sensitivity and specificity of four questions (HARK) to identify intimate partner violence: a diagnostic accuracy study in general practice

**DOI:** 10.1186/1471-2296-8-49

**Published:** 2007-08-29

**Authors:** Hardip Sohal, Sandra Eldridge, Gene Feder

**Affiliations:** 1 Centre for Health Sciences, Barts and the London, Queen Mary's School of Medicine and Dentistry, 2 Newark Street, London, E1 2AT, UK

## Abstract

**Background:**

Intimate partner violence (IPV) including physical, sexual and emotional violence, causes short and long term ill-health. Brief questions that reliably identify women experiencing IPV who present in clinical settings are a pre-requisite for an appropriate response from health services to this substantial public health problem. We estimated the sensitivity and specificity of four questions (HARK) developed from the Abuse Assessment screen, compared to a 30-item abuse questionnaire, the Composite Abuse Scale (CAS).

**Methods:**

We administered the four HARK questions and the CAS to women approached by two researchers in general practice waiting rooms in Newham, east London. Inclusions: women aged more than 17 years waiting to see a doctor or nurse, who had been in an intimate relationship in the last year. Exclusions: women who were accompanied by children over four years of age or another adult, too unwell to complete the questionnaires, unable to understand English or unable to give informed consent.

**Results:**

Two hundred and thirty two women were recruited. The response rate was 54%. The prevalence of current intimate partner violence, within the last 12 months, using the CAS cut off score of ≥3, was 23% (95% C.I. 17% to 28%) with pre-test odds of 0.3 (95% C.I. 0.2 to 0.4). The receiver operator characteristic curve demonstrated that a HARK cut off score of ≥1 maximises the true positives whilst minimising the false positives. The sensitivity of the optimal HARK cut-off score of ≥1 was 81% (95% C.I. 69% to 90%), specificity 95% (95% C.I. 91% to 98%), positive predictive value 83% (95% C.I. 70% to 91%), negative predictive value 94% (95% C.I. 90% to 97%), likelihood ratio 16 (95% C.I. 8 to 31) and post-test odds 5.

**Conclusion:**

The four HARK questions accurately identify women experiencing IPV in the past year and may help women disclose abuse in general practice. The HARK questions could be incorporated into the electronic medical record in primary care to prompt clinicians to ask about recent partner violence and to encourage disclosure by patients. Future research should test the effectiveness of HARK in clinical consultations.

## Background

Violence against women is a global issue affecting millions who experience it and have to live with its consequences [1]. Intimate partner violence (IPV) including physical, sexual and emotional abuse is a major public health problem.

The WHO Violence Against Women study [2] found that the prevalence of lifetime physical violence and sexual violence by an intimate partner, among ever-partnered women varied from 15 to 71% in urban and rural settings in 10 countries. The prevalence of IPV is higher among women seeking primary care than in community surveys of the same geographic populations [3].

In a study in 12 east London general practices it was found that 41% of women waiting to see their general practitioner (GP) or practice nurse had experienced physical violence from a partner or former partner. 17% had experienced it within the past year [4].

IPV causes short and long term health problems. From controlled studies in a wide range of settings, we know that these include injury, chronic pain, gastrointestinal and gynaecological conditions (including sexually transmitted diseases) [5]. Consequences of IPV extend to perinatal health with it being an independent risk factor for deficit in gestational weight gain during pregnancy [6] and strong evidence of an IPV association with low birth weight [7].

The psychological health problems associated with domestic violence are no less serious and have psychological parallels with the trauma of being taken hostage and subjected to torture [8]. The most prevalent mental health sequelae of IPV are depression and post-traumatic stress disorder [9].

Women who have experienced physical or psychological violence are fifteen times more likely to abuse alcohol and nine times more likely to abuse drugs than are non-abused women, and there is evidence that substance abuse is a consequence as well as a potential cause of IPV [10]. Children exposed to domestic violence also often experience emotional and behavioural problems [11]. In the developing world it has been shown that children exposed to severe and recurrent IPV are more likely to be admitted with severe acute malnutrition [12].

It is difficult to calculate the exact societal economic impact of IPV but the costs are high. In the United States annual costs of intimate partner rape, physical assault, and stalking exceed $5.8 billion, nearly $4.1 billion of which is for direct medical and mental health care services [13]. In the United Kingdom the annual cost to the national health service of physical assaults is £1.2 billion [14].

The Department of Health in England now recommends that "All trusts should be working towards routine enquiry" [15]. In the US, the Family Violence Prevention fund consensus guidelines recommend that all adolescent and adult patients should be routinely asked about domestic violence [16]. Although there is ongoing debate about the evidence for screening or routine enquiry [17], there is unquestionably a need for clinicians to ask about domestic violence more often than they currently do.

A study of women attending general practices in east London found that only 17% of women experiencing IPV reported that their doctor had asked them about domestic violence [4]. We know that women who are experiencing violence want to disclose this to trusted doctors and get support [18], but that a high proportion of women who are experiencing abuse do not disclose this spontaneously in clinical consultations [4].

Short questions that reliably identify women experiencing IPV who present in clinical settings are a pre-requisite for developing an appropriate response from health services to this substantial public health problem [19].

Many primary health care professionals, including general practitioners (GPs) and practice nurses, occasionally enquire about domestic violence. It has not been adequately determined whether their questions identify women experiencing IPV.

### Short tests

We have identified eleven short tools (see Additional file 1), for identification of women experiencing IPV [20-30]. Only three were validated in primary care settings [20-22]. The first study did not consider sexual abuse and had an unrepresentative sample: it was able to differentiate between self identified survivors of abuse and non-abused patients; there was no evidence that it was able to identify women who had experienced IPV in a general practice population [20]. The second reported no sensitivity or specificity; instead there was correlation between their tool and the reference test (Abuse Risk Inventory, r = 0.69, p = 0.01) but this does not necessarily indicate a valid and specific measure of IPV [21]. The third tool, a single question about safety, had low sensitivity, positive and negative predictive values (9%, 63% & 57% respectively) [22].

Outside of primary care settings, another two instruments did not consider sexual abuse [23,24]. One reported no sensitivity or specificity; only those who were positive on the index test were recruited into the study [23]. The sensitivity, specificity and positive predictive value of the other test were too low for use by clinicians (65%, 80% & 51% respectively) [24].

The sixth tool [25] had a low positive predictive value: 56%. The seventh instrument started with an open question which makes it difficult to use as a standardised tool [26] and the eighth, Webster's "self-report check list," was not validated against an appropriate reference standard so there was no calculation of test indices [27]. Two further studies evaluated single item measures [28,29] and concluded that these may not be adequate in assessing for domestic violence.

We believe that the eleventh instrument, the AAS [30] has the most potential. Its strengths include that it covers a wide definition of partner violence which includes sexual abuse; a number of the aforementioned tools do not include sexual abuse [20,23,24,28]. It has 5 items rather than an unsatisfactory single item as is the case with a number of the tools [22,28,29]. Additionally it has a simple scoring system which we believe is important in brief general practice consultations unlike the likert scales used in 2 of the tools [20,25], the multiple scoring protocols in one [21] and an open question in another [26]. Finally, it has also been validated against an appropriate reference standard, the Index of Spouse Abuse (ISA) [31] unlike some [27].

However we also feel that the AAS has a number of weaknesses. Although the investigators concluded that the AAS questions were valid, this was based on a correlation between the score on a three-question version of the AAS and the ISA. No sensitivity or specificity was reported. Furthermore, the AAS validation was only within the setting of antenatal care in the US [30]. We do not know whether this is generalisable to other health care settings and in other countries, preventing its implementation into UK clinical practice [32].

More recently, in 2004, the test performance of the AAS was evaluated against the modified version of the conflict tactics scale (CTS 2) [33]. The AAS's sensitivity for minor physical violence was 32% and for severe physical violence was 61%. It was concluded that it was not sensible to use the AAS as a screening tool until more evidence was gathered.

In our study we have adapted the AAS, for use in a general practice setting, to form the HARK questionnaire (see table 1). We tested the HARK against the 30-item Composite Abuse Scale (CAS, see table 2) [34].

**Table 1:** HARK questions – one point is given for every yes answer

**H**	HUMILIATION
	Within the last year, have you been humiliated or emotionally abused in other ways by your partner or your ex-partner?
**A**	AFRAID
	Within the last year, have you been afraid of your partner or ex-partner?
**R**	RAPE
	Within the last year, have you been raped or forced to have any kind of sexual activity by your partner or ex-partner?
**K**	KICK
	Within the last year, have you been kicked, hit, slapped or otherwise physically hurt by your partner or ex-partner?

**Table 2:** Dimensions and items of the Composite Abuse Scale

Severe combined abuse	Kept me from medical care
	Used a knife or gun or other weapon
	Locked me in the bedroom
	Put foreign objects in my vagina
	Refused to let me work outside the home
	Raped me
	Tried to rape me
	Took my wallet and left me stranded
	
Emotional abuse	Told me that I was crazy
	Tried to convince family, friends and children that I was crazy
	Became upset if dinner/housework wasn't done when they thought it should be
	Told me that I wasn't good enough
	Tried to keep me from seeing or talking to my family
	Told me that I was stupid
	Tried to turn my family, friends and children against me
	Did not let me socialise with my female friends
	Told me that I was ugly
	Told me no one would ever want me
	Blamed me for their violence
	
Physical abuse	Shook me
	Hit or tried to hit me with something
	Pushed, grabbed or shoved me
	Kicked me, bit me or hit with a fist
	Slapped me
	Threw me
	Beat me up
	
Harassment	Harassed me over the telephone
	Harassed me at work
	Followed me
	Hung around outside my house

## Methods

We conducted a cross-sectional survey of women in GP waiting rooms. The fifty-one general practices in Newham, a multi-ethnic inner city area of London, were stratified according to the number of doctors and the proportion of south Asian names on the practice register [35]. Equal numbers of practices were selected from each stratification group using a randomisation programme (SPSS version X). This was in an attempt to ensure that the practice population reflected the local area population.

Each practice was sent a recruitment letter with information about the study. If practices expressed an interest, a research team member met with the primary care team to answer any questions. We excluded practices that did not have a private room available, as then privacy for the survey could not be provided. If a practice decided not to take part or was excluded, the reason for this was documented and another practice was randomly selected from within the same stratification group.

We approached consecutive women in practice reception areas waiting to see a doctor or nurse. We included women aged more than 17 years who in the last year had been in an intimate relationship. We excluded women who were accompanied by children over four years of age or by another adult, were too unwell to complete the questionnaires, unable to understand English or unable to give informed consent. In the waiting room, women were asked to participate in a study designed to improve women's health care. We sought consent for the administration of the HARK and CAS questionnaires in a private room. All participants were given information on local domestic violence services. The East London and City ethics committee approved the study.

The number of potentially eligible subjects was recorded by the researcher in the waiting room. A record was made of the number of women who were excluded due to the exclusion criteria, those who the researchers were unable to approach at very busy times, women who were approached and agreed that they would be seen by the clinician first and then undertake the study but were not seen again ("did not come back"), those who refused participation in the waiting room and those who declined consent in the private room.

The HARK and CAS were self-administered. We expected to be able to recruit approximately 500 women. On the assumption that the prevalence of IPV in the past year was 20%, we calculated that there was a 90% chance of estimating sensitivity at 76% or above with this sample size.

### The Composite Abuse Scale – the reference standard

The CAS is a relatively robust standard for identifying IPV in primary care settings. It has an internal reliability (Cronbach's alpha) of .90 or more for each sub-scale, and all item-total score correlations of .6 or above [34]. It has also been validated with a large (1,836) sample of patients in general practice settings [36]. It is based on a concept of IPV that includes coercion, not simply violent acts arising out of conflict. It is recommended as an IPV research assessment tool by the National Centre for Injury Prevention and Control [37], as it has demonstrated reliability and validity for measuring the self-reported incidence and prevalence of IPV. It has evidence of content, construct, criterion and factorial validity. The CAS measures four dimensions of abuse inflicted on a woman by her partner: physical abuse (PA), emotional abuse (EA), severe combined abuse (SCA) and harassment. A preliminary cut-off score of 3 divides women presenting as abused or non-abused in general practice settings [36]. The 30 items are listed in table 2.

### HARK – the index test

The acronym HARK denotes four short questions which represent different components of IPV. "Hark" is an archaic verb that means "to listen attentively." HARK arose out of an adaptation of the AAS. In HARK there is a focus only on IPV (not including that committed by a stranger), the pregnancy related item has been removed (so that it can be used in all women), for clarity emotional and physical violence are separated out into 2 items (rather than being combined in 1), "humiliation" was added (as it was thought to be plainer English and have a wider remit then "emotional abuse"), "rape" was added (to try to help cue a woman's memory by using language similar to her own) whilst items relating to fear and physical violence were directly retained from the AAS. The HARK questions are listed in table 1.

None of the women who were identified as having suffered abuse requested the researcher to make a direct referral in order to access specialised services.

### Outcomes measures

The rate of current IPV within the last twelve months was calculated for the CAS (using the cut off score of ≥3) with 95% confidence intervals. This is equal to the prevalence or pre-test probability of IPV within the last twelve months.

The rates of IPV within the last twelve months were also calculated with 95% confidence intervals for the HARK, at different cut off scores (e.g. HARK cut off score ≥2, means a HARK score of either 2, 3 or 4). Each woman was identified as being positive or negative for IPV for each HARK cut off score and for the CAS cut off score of ≥3. We could then calculate HARK's sensitivity, specificity, positive predictive value (PPV – also known as the post-test probability), negative predictive value (NPV), likelihood ratios (LRs) with 95% confidence intervals and post-test odds (= pre-test odds × LR) at different HARK cut off scores [38].

A receiver operator characteristic (ROC) curve was constructed by plotting the sensitivity of each different HARK cut off against the false positive rate (= 100 - specificity) at the different HARK cut offs. This was used to determine an optimal cut off HARK score which maximised the true positives whilst minimising the false positives.

Multilevel LRs [38] were also calculated at different HARK scores (e.g. a HARK score of 2 means 2 only, not ≥2, i.e. 2, 3 or 4) with 95% confidence intervals and corresponding post-test odds. 95% confidence intervals were calculated in EXCEL. Multilevel LRs allow exploration of the diagnostic usefulness of individual HARK scores.

We have used a variety of different methods to assess HARK's diagnostic accuracy at identifying IPV. Sensitivity and specificity interpret the HARK results retrospectively whereas PPVs and NPVs establish the predictive properties of the HARK in the future. The PPV is the proportion of women with a specific HARK result who are experiencing IPV. LRs express a result in terms of the actual chances of a woman experiencing IPV if her HARK score reaches a particular level. A LR for a given HARK result gives the odds that the test result comes from a person who is experiencing IPV. Unlike PPV and NPV, LRs are a good deal more constant with changes in prevalence. The post test odds allow background prevalence to be factored into the LR. Multilevel LRs express HARK's accuracy with level-specific likelihood ratios. They can be calculated at different HARK scores (e.g. 1) as opposed to cut offs (e.g. ≥1). They ensure that the maximum information is derived from the total range of possible HARK results (0 to 4).

## Results

**Figure 1:**
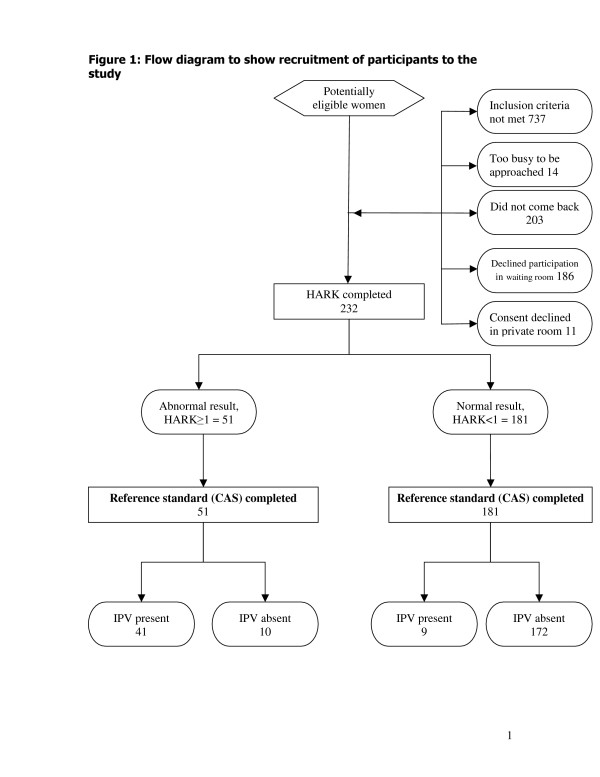
Flow diagram to show recruitment of participants to the study.

We approached 24 practices and 12 agreed to participate; 11 declined and one was excluded as it had no private room. Two hundred and thirty two women were recruited from May to October 2003. Figure 1 shows recruitment of individual participants to the study. Seven hundred and thirty seven women did not meet the inclusion criteria. Fourteen women were not approached because there were too many women in the waiting room for all to be approached. Two hundred and three women "did not come back." One hundred and eighty six women declined participation in the waiting room. Eleven women declined consent in the private room. The response rate of 54% (232/(232 + 186 + 11) was adjusted for the women who "did not come back."

The average age of participants was 35 years (range 18–70 years). 51% were in a paid job and 53% owned a house or flat. 40% of participants described their ethnic origin as white British, 25% as black British, African or Caribbean and 18% as Indian, Pakistani or Bangladeshi.

### Outcomes measures

The CAS identified 53 cases of current IPV in the study population. This produced a prevalence (pre-test probability) of current IPV of 23% (95% C.I. 17% to 28%) with pre-test odds of 0.30 (95% C.I. 0.23 to 0.38). Pretest odds are prevalence divided by one minus prevalence.

**Figure 2:**
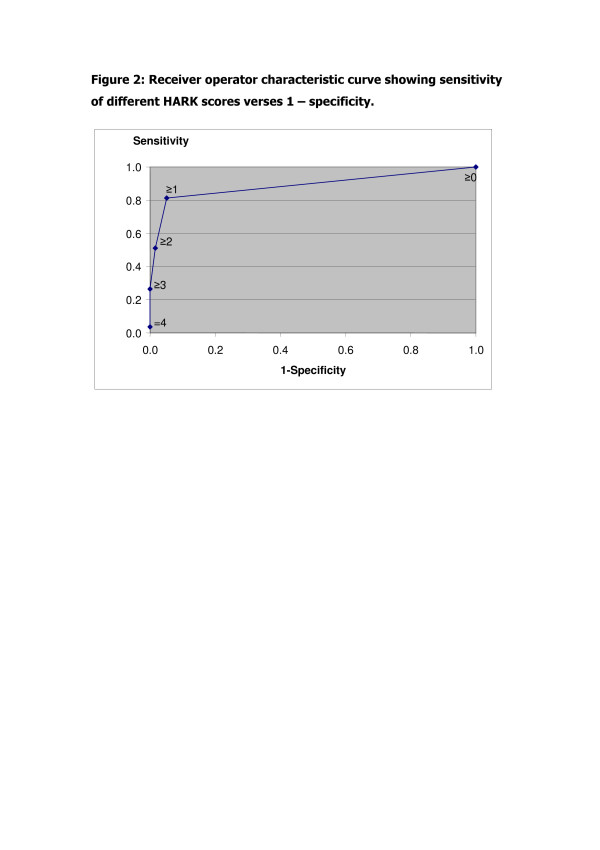
Receiver operator characteristic curve showing sensitivity of different HARK scores verses 1 - specificity.

Table 3 gives the sensitivity, specificity, PPV, NPV, LRs and post-test odds of HARK at different cut off scores. The receiver operator characteristic curve (figure 2) demonstrated that a HARK score ≥1 is the optimal cut off for detecting IPV. The predictive properties of the HARK score of ≥1 are highlighted in table 3. The HARK test accuracy (using a cut-off of ≥1) is 92%. This represents the proportion of true positives and true negatives as a proportion of all results.

**Table 3:** The sensitivity, specificity, PPV, NPV, LR & post-test odds with 95% confidence intervals of HARK at different cut off scores

**Hark cut off scores**	**% of study sample**	**Sensitivity with 95% C.I.**	**Specificity with 95% C.I.**	**Positive predictive value with 95% C.I.**	**Negative predictive value with 95% C.I.**	**Likelihood ratio with 95% C.I.**	**Post-test odds**
= 4	1%	4% (3% to 13%)	100% (98% to 100%)	100% (22 to 100%)	78% (72% to 83%)	Undefined	Undefined
≥3	6%	26% (15% to 40%)	100% (98% to 100%)	100% (81% to 100%)	82% (76% to 87%)	Undefined	Undefined
≥2	13%	51% (37% to 65%)	98% (95% to 100%)	90% (73% to 98%)	87% (82% to 91%)	30 (10 to 96)	9
≥1	22%	81% (69% to 90%)	95% (91% to 98%)	83% (70% to 91%)	94% (90% to 97%)	16 (8 to 31)	5
≥0	100%	100% (93% to 100%)	0% (0% to 2%)	23% (18% to 29%)	error	1	0.3

When the specificity is 100%, the likelihood ratio and post test odds are undefined.

Confidence intervals for likelihood ratios are approximate since they were calculated by the delta method which is less reliable when some cell sizes are small (Armitage P, Matthews JNS, Berry G. *Statistical methods in medical research*. Oxford; Blackwell, 1994).

Multilevel LRs calculated at different HARK scores with 95% confidence intervals and corresponding post-test odds are shown in table 4.

**Table 4:** Multilevel likelihood ratios with 95% confidence intervals and post-test odds of individual HARK scores.

**HARK score (number of "yeses")**	**Likelihood ratio with 95% C.I.**	**Post-test odds**
3 or 4	Undefined	Undefined
2	14.6 (4.3 to 49.4)	4.3
1	9.01 (3.7 to 21.9)	2.67
0	0.2 (0.1 to 0.4)	0.1

## Discussion

The four HARK questions accurately identify women experiencing IPV in the past year and may help women disclose IPV in general practice. The estimated specificity (95%, 95% C.I. 91% to 98%) of the HARK score of ≥1 was higher than the sensitivity (81%, 95% C.I. 69% to 90%). The PPV (post-test probabilities) of HARK, which increase as the HARK score increases, also provide evidence that HARK is an effective short tool for identifying IPV.

The most straightforward way of using the HARK is as a simple test with a cut off of ≥1. Therefore if a clinician asks these four questions and their patient scores ≥1, this will identify 81% of women affected by IPV (as judged by the CAS). This is assuming that the tool performs in the same way that it did when a researcher administered it. There is an 83% probability that a woman with this score has experienced IPV in the past year (positive predictive value); and she is 16 times more likely to have been affected by IPV in the last year than some-one with a HARK score of 0 (likelihood ratio of a positive result).

The multilevel LRs and corresponding post-test odds make more use of the data from the test as it avoids dichotomising the HARK score into IPV present or not present [39]. When a woman is asked the four HARK questions she does not actually have a positive or negative score for IPV; instead she may score 0, 1, 2, 3 or 4 and each score has a different meaning (i.e. different likelihood ratio and post-test odds for IPV – see table 4). When an individual answers "no" to all of the HARK questions the likelihood ratio and post test odds (0.2 and 0.1 respectively) suggest that IPV is probably not present; whereas answering "yes" to three or four HARK questions produces a specificity of 100%, meaning that IPV is present. Answering "yes" to one or two of the HARK questions is less specific.

The majority of women who are experiencing IPV do not spontaneously disclose to clinicians. HARK can potentially accurately and quickly identify a high proportion of these women. This is a pre-requisite for effective intervention allowing the successful management of IPV in general practice. It has been shown that women want to disclose IPV to health care professionals, particularly primary care clinicians [18].

The high pre-test probability (prevalence) of IPV (23%) is consistent with other prevalence studies in primary health care settings [3].

To increase the external validity of the study, we recruited a wide range of practices, including small single handed ones with less than 3,000 patients which are common in inner city areas in the United Kingdom. However small practices had fewer patients in the waiting room available for recruitment than had been anticipated; with the recruitment of participants taking longer than planned. Consequently we were only able to recruit 46% of our target sample size within the timeframe of the study, resulting in less precise estimates of test accuracy, reflected in wider confidence intervals. Nevertheless our study is larger than some other validation studies of short instruments and our estimates of test characteristics are relatively precise.

Eighty two percent of women who did not fulfil the inclusion criteria were accompanied. The ethics committee that approved our proposal specified that potential participants should only be approached if they were unaccompanied in order to decrease the likelihood of an abusive partner discovering that the participant had completed a questionnaire on domestic violence. We did include women who were accompanied by children under the age of five years, as it was felt that a child this young was unlikely to jeopardise a participant's safety.

Overall women were enthusiastic about participation once they found out that the study was about domestic violence: only eleven women declined consent in the private room. One hundred and eighty six women declined participation in the waiting room but these women did not know that the study was specifically about domestic violence.

The National Census 2001 figures allowed us to compare our study population to the local population in the borough of Newham. The average age of the study population was 3 years older than the average age in the local population (32 years). The percentage of the study population in a paid job was 12% higher and the percentage that owned a house or flat was 9% higher than that in the local population (39% and 44% respectively). The percentage of the study population that described their ethnic origin as white British was 6% higher than that in the local population (34%) whilst the percentage that described their ethnic origin as Indian, Pakistani or Bangladeshi was 11% lower than that in the local population (29%). This comparison shows that despite our attempts, the study population were not totally representative of the local population. We believe that the higher socio-economic status of our study sample (as reflected by the higher percentage in a paid job and owning a house or flat) compared to the local population may reflect a response bias meaning that perhaps those women with lower socio-economic status and at greater risk of IPV were less likely to have taken part in this study. This may have affected the calculation of the prevalence, PPV and NPV of HARK. However there is no reason why this would necessarily affect the sensitivity/specificity calculations unless the 46% of women who did not take part in the study answered differently with regards to only one of the instruments (the HARK or the CAS). This is unlikely.

The strengths of this study are that it tested a short tool that can be used in routine general practice, against an abuse measure validated in primary care. Additionally, HARK's external validity has been increased by being conducted in a range of practices with a study population of varied ethnicity.

Limitations included the response rate of 54%, decreasing the external validity of the study. Although we consider the CAS to be the best research measure for IPV in a health care setting, we cannot exclude the possibility that not all women who were found to be positive for IPV with the HARK but negative with the CAS were false positives. Other investigators have found that when using two sets of validated questions each may identify some women as abused that would have been missed by the other tool [40].

The HARK questions could be incorporated into electronic medical records in primary care to prompt clinicians to ask about recent intimate partner violence and to encourage disclosure by patients. Future research should test the effectiveness of HARK in clinical consultations as part of system level interventions to improve the response of primary care to IPV.

## Conclusion

Intimate partner violence against women is common and causes short and long-term ill health. Previously questions about intimate partner violence to elicit disclosure have been insufficiently validated for use in general practice or family medicine populations, particularly outside the US. The four short HARK questions accurately identify women experiencing intimate partner violence in the past year.

## Competing interests

The author(s) declare that they have no competing interests.

## Authors' contributions

GF had the original idea of the study which was then developed by HS. SE advised on sample size and analysis. Guarantors: HS and GF.

All authors read and approved the final manuscript.

## Pre-publication history

The pre-publication history for this paper can be accessed here:



## Supplementary Material

Additional file 1
