# Supplementary material for: The sensitivity and specificity of four questions (HARK) to identify intimate partner violence: a diagnostic accuracy study in general practice
Source: BMC Fam Pract. 2007 Aug 29;8:49. doi: 10.1186/1471-2296-8-49 (PMC2034562; doi:10.1186/1471-2296-8-49)
Supplement: Additional file 1 [file 1471-2296-8-49-S1.doc]

**Table 1: Eleven short measures for detecting intimate partner violence in health care settings**

| **Paper** | **Evaluated**  **tools** | **Setting** | **Prevalence** | **Reliability** | **Concurrent validity** | **Sensitivity** | **Specificity** | **Positive**  **Predictive**  **Value** | **Negative**  **Predictive**  **Value** | **Likelihood**  **Ratio** |
| --- | --- | --- | --- | --- | --- | --- | --- | --- | --- | --- |
| Brown, Lent, Schmidt &  Sas (2000)^21^ | **Index:** Woman  Abuse Screening  Tool (WAST)  **Comparator:**  Abuse Risk  Inventory (ARI) | Canada  Family practice site | Period  WAST -  8.5% | Coefficient  alpha- 0.75 | Correlation  between WAST  & ARI:  r= 0.69,  p=0.01 | - | - | - | - | - |
| Connelly, Newton,  Landsverk & Aarons  (2000)^29^ | **Index:** Single  question hospital  screen “Are you in  a relationship in  which you have  been threatened,  scared or hurt by  someone?” If yes,  whom?  **Comparator:**  Conflict Tactics  Scale (CTS) | Not entirely clear where tools were administered i.e.  at hospital or home visit. | Single  question- 4%  CTS- 18.6% | Not stated | Not stated | - | - | - | - |  |
| Ernst, Weiss, Cham, Hall  & Nick (2004)^25^ | **Index:** Ongoing  Violence  Assessment Tool  (OVAT)  **Comparator:**  Index of Spouse  Abuse (ISA) | USA  A&E department | OVAT- 33%  ISA- 21% | Cronbach’s  alpha= 0.6*  Kappa= 0.58  (95%CI 0.53-  0.63)*  * Based on  male and  female data | Inter-item  correlation of  0.38 (variance  0.03)*  *Based on male  and female data | 86 | 83 | 56 | 96 | 5 |
| Feldhaus, Kozioi-McLain,  Amsbury, Norton,  Lowenstein & Abbott  (1997)^24^ | **Index:** Partner  Violence screen  (PVS)  **Comparator:**  Index of Spouse  Abuse (ISA)  Conflict Tactics  Scale (CTS) | USA  Two urban, hospital-based A&E departments | Current  PVS- 29.5%  (95% CI:  24.6%-  34.8%)  ISA- 24.3%  (95% CI:  19.2%-  30.1%)  CTS- 27.4%*  (95%CI:  21.7%-  33.6%). | Not stated | Not stated | Vs ISA  65  Vs  CTS  71 | 80  84 | 51  63 | 88  89 | 3  5 |
| Heron , Thompson, Jackson, Kaslow (2003)^23^ | **Index:**  Universal Violence Prevention Screening Protocol.  Only those who were positive on the index test were recruited into the study | USA  A&E department | - | - | - | - | - | - | - | - |
| McFarlane, Parker,  Soeken, & Bullock  (1992)^30^ | **Index:**  Abuse Assessment  Screen (AAS)  **Comparator:**  Conflict Tactics  Scale (CTS)  Index of Spouse  Abuse (ISA) | USA  Two Perinatal clinics | ASS:  Physical and  sexual  violence  Within last  year  26%  During  pregnancy  17%  Combined  55% | Not stated | Not stated | - | - | - | - | - |
| Peralta & Fleming  (2003)^22^ | **Index:**  1 question: “Do  you feel safe at  home?”  **Comparator:**  Modified Conflict  Tactics Scale  (CTS) (6 items) | USA  Urban family practice clinic | Period (90  days)  CTS- 44.3% | Not stated | Not stated | 9 | 96 | 63 | 57 | 2 |
| Reid, Biringer, Carroll, Midmer, Wilson, Chalmers, Stewart (1998)^26^ | **Index:** Antenatal Psychosocial Health Assessment (ALPHA) form.  Starts with an open question - difficult to use as a standardised tool  **Comparator:**  Nil | Various urban and rural locations across Ontario, providing antenatal care | - | - | - | - | - | - | - | - |
| Sagrestano, Rodriguez,  Carroll, Bieniarz,  Greenberg, Castro, &  Nuwayhid (2002)^28^ | **Index:**  Perinatal Self-  Administered  Inventory (PSAI)-  contains two  questions on DV  **Comparator:**  Conflict Tactics  Scale (CTS) | Mid-Western USA  University affiliated women’s care centre | Verbal  aggression-  84.3%, 17%  experienced  violence in  past year.  During the  current  pregnancy,  verbal abuse-  68.1%,  violence  13.3% | Not stated | PSAI- “Are you  suffering mental  or physical  abuse now?”  and  • Standard  scale verbal  aggression  (CTS)  r=.03  (p>0.05)  • Standard  scale violence  (CTS)  r=.05 (p>0.05) | - | - | - | - | - |
| Webster, Holt (2004)^27^ | **Index:**  Self report check list.  **Comparator:**  Not validated against an appropriate reference standard | Prenatal clinic in Australia | - | - | - | - | - | - | - | - |
